# The incidence of depression and anxiety in patients with ankylosing spondylitis: a systematic review and meta-analysis

**DOI:** 10.1186/s41927-019-0111-6

**Published:** 2020-03-02

**Authors:** Jamie YE. Park, Alyssa M. Howren, Enav Z. Zusman, John M. Esdaile, Mary A. De Vera

**Affiliations:** 1 Faculty of Pharmaceutical Sciences, University of British Columbia, 2405 Wesbrook Mall, Vancouver, BC V6T 1Z3 Canada; 2 Collaboration for Outcomes Research and Evaluation, Vancouver, BC Canada; 3 Arthritis Research Canada, Vancouver, BC Canada

**Keywords:** Ankylosing spondylitis, Depression, Anxiety, Mental health, Systematic review, Meta-analysis

## Abstract

**Background:**

As awareness for the importance of mental health continues to expand in rheumatology, it is important to understand the epidemiology of psychiatric complications in ankylosing spondylitis (AS) with the ultimate goal of future prevention and improved quality of care. This study aims to review evidence on the incidence and determinants of depression and/or anxiety among patients with AS.

**Methods:**

We searched Medline, Embase, Cochrane Database of Systematic Reviews, CINAHL Complete, and PsycINFO for full-length observational studies that involved a sample or population of patients with AS and assessed depression and/or anxiety. Primary outcomes extracted were: 1) risk estimates for depression and/or anxiety (e.g., relative risk [RR]); and 2) determinants or factors identified as independent predictors of depression and/or anxiety using multivariable regression approaches and corresponding estimates (e.g., odds ratios [OR]). Where relevant, we pooled estimates using random effects models.

**Results:**

Out of 783 titles from our search strategy, we reviewed 39 manuscripts. Four studies assessed the incidence of depression and meta-analyzing reported estimates from three of these studies yielded a pooled RR of 1.51 (95% CI 1.28 to 1.79). Differences in risk of depression among men and women with AS were inconclusive, suggesting need for further study. The incidence of anxiety was comparatively less studied with only one included study reporting a hazard ratio of 1.85 (95% CI 1.37 to 2.49). Education level was a key determinant, with lower levels associated with higher odds of depression (OR 6.65; 9% CI 1.36 to 32.51) and anxiety (OR 9.31; 9% CI 1.39 to 62.19) among AS patients.

**Conclusions:**

Our systematic review and meta-analysis shows an increased risk of depression and anxiety among patients with AS. These findings suggest the importance of monitoring and care for psychiatric conditions in AS.

## Background

Ankylosing spondylitis (AS) is an inflammatory arthritis that mainly affects the axial skeleton and is characterized by inflammatory back pain and progressive bony fusion of the vertebral column [1]. Arthritis in the hips, shoulders, and/or peripheral joints may also occur [2]. The presence of radiographic sacroiliitis differentiates patients with AS from those with non-radiographic spondyloarthritis, and both of these diagnoses are collectively referred to as axial spondyloarthritis according to the 2009 classification criteria from the Assessment of SpondyloArthritis International Society [3]. AS is 2 to 3 times more common in males compared to females [4–6] and prevalence reports for AS range from 0.1% up to 1.4%, making it one of the most common of inflammatory arthritides [1, 2]. AS is also a young-onset disease, with initial symptoms occuring before the age of 40 in about 90% of patients [7]. Along with its physical impacts on patients’ pain and disability [8–10], studies have also shown the association of AS with physical complications including various cardiovascular diseases [11, 12].

Equally important to physical impacts are the psychological impacts of AS, particularly with evidence syntheses showing substantial prevalence of depression among patients. Specifically in 2016, Hopkins et al. [13] presented an abstract on their systematic review of 17 cross-sectional studies on the prevalence of depression in AS, reporting prevalence estimates ranging from 4.9 to 55.5%. More recently in 2018, Zhao et al. [14] systematically reviewed 15 epidemiologic studies reporting an overall pooled prevalence proportion of 0.29 (95% confidence interval [CI]) 0.15 to 0.44) and comparable burden of depression among cohorts with axial spondyloarthritis and non-radiographic axial spondyloarthritis. With prior systematic reviews largely including cross-sectional studies and focusing on prevalence, there is a need to better understand incidence, that is, the risk of developing depression after AS diagnosis. As awareness for the importance of mental health continues to expand in rheumatology, it is also important to evaluate other psychiatric conditions. As such, to comprehensively understand the epidemiology and determinants of psychiatric complications in AS with the ultimate goal of future prevention and improved quality of care, we conducted a systematic review and meta-analysis with the following objectives: 1) determine the incidence of depression and/or anxiety in AS; and 2) identify factors associated with depression and/or anxiety among patients with AS.

## Methods

### Literature search strategy

Our systematic review was guided by the Preferred Reporting Items for Systematic Reviews and Meta-Analysis (PRISMA) guidelines [15]. The search strategy was developed in collaboration with an information scientist who then applied it to health-related databases with international coverage in April 2018. These databases included Medline, Embase, and Cochrane Database of Systematic Reviews on the Ovid platform, and CINAHL Complete and PsycINFO on Ebscohost. Where subjects are well-indexed, subject headings were used to increase relevance and precision of search results and to ensure a manageable number of items retrieved; where subjects are less well-indexed, or had not yet been assigned subject headings, key words were added to increase recall. Subject headings used were database dependent, but analogous to the Medical Subject Headings (MeSH) used in Medline. The search strategy is provided in Additional file 1.

### Study selection and eligibility criteria

Titles and abstracts were reviewed for articles meeting the following criteria: 1) observational study (e.g., cross-sectional, case-control, cohort study) published as a full-length article; 2) study sample or population of patients with AS, with or without a control group of individuals without AS; 3) depression and/or anxiety assessed using routinely reported measures (e.g., self-report, clinical diagnosis, International Classification of Diseases [ICD] codes, and validated questionnaires such as the Hospital Anxiety and Depression Scale [HADS]); 4) reporting of relevant estimates (e.g., relative risk [RR] for incidence of depression and/or anxiety, odds ratio [OR] for studies assessing determinants of depression/anxiety or ratio); and 6) published in English. We excluded conference abstracts, posters, and grey literature (including theses). Abstracts that met our inclusion criteria were then reviewed in full text to form a final list of included studies.

### Data extraction, outcomes, and quality assessment

We extracted the following information from included studies: year of publication, country, study design, study setting (e.g., outpatient) or data source (e.g., administrative health database), sample size, participants’ characteristics (i.e., age, gender), methods for diagnosing AS, and methods for assessing depression and anxiety. Primary outcomes of interest included both reported incidence estimates for depression and/or anxiety and determinants of depression and/or anxiety in AS, that is, factors that are independently associated with these conditions, as shown in multivariable regression models. For the latter, we extracted information on the determinant (e.g., smoking) along with reported risk estimates, namely adjusted ORs and corresponding 95% confidence intervals (CI). Given that parameters of our search and eligibility screening would capture studies of the prevalence of depression and/or anxiety in AS, as secondary outcomes, we extracted relevant estimates (e.g., prevalence proportion, OR) or where possible, or calculated these based on reported information. Finally, we assessed the quality of included studies using the Newcastle-Ottawa Scale for cohort and case-control studies [16] and the modified Newcastle-Ottawa Scale for cross-sectional studies [17]. There are three sections that both of these questionnaires assess, namely, the selection of the groups, comparability of the groups, and the exposure or outcome of the groups [18]. We have adapted the threshold for quality assessment for the purposes of the study. The Newcastle-Ottawa Scale is out of maximum 8 points and the study is considered to have good quality if the overall score falls within 6 to 8 points (≥3 points in selection, 1 point in comparability, ≥2 points in outcome/exposure) [17, 18]. The modified Newcastle-Ottawa Scale is out of maximum 9 points and the study is considered to have good quality if the overall score falls within 7 to 9 points (≥4 points in selection, 1 point in comparability, ≥2 points in outcome/exposure) [17, 18].

### Synthesis

We conducted a narrative synthesis of findings from included studies. As well, we meta-analyzed reported incidence estimates for depression and anxiety using random-effect models. We used the I^2^ as a measure of heterogeneity. We performed all data analysis using Stata V. 14 (StataCorp, College Station, TX, USA).

## Results

**Fig. 1:**
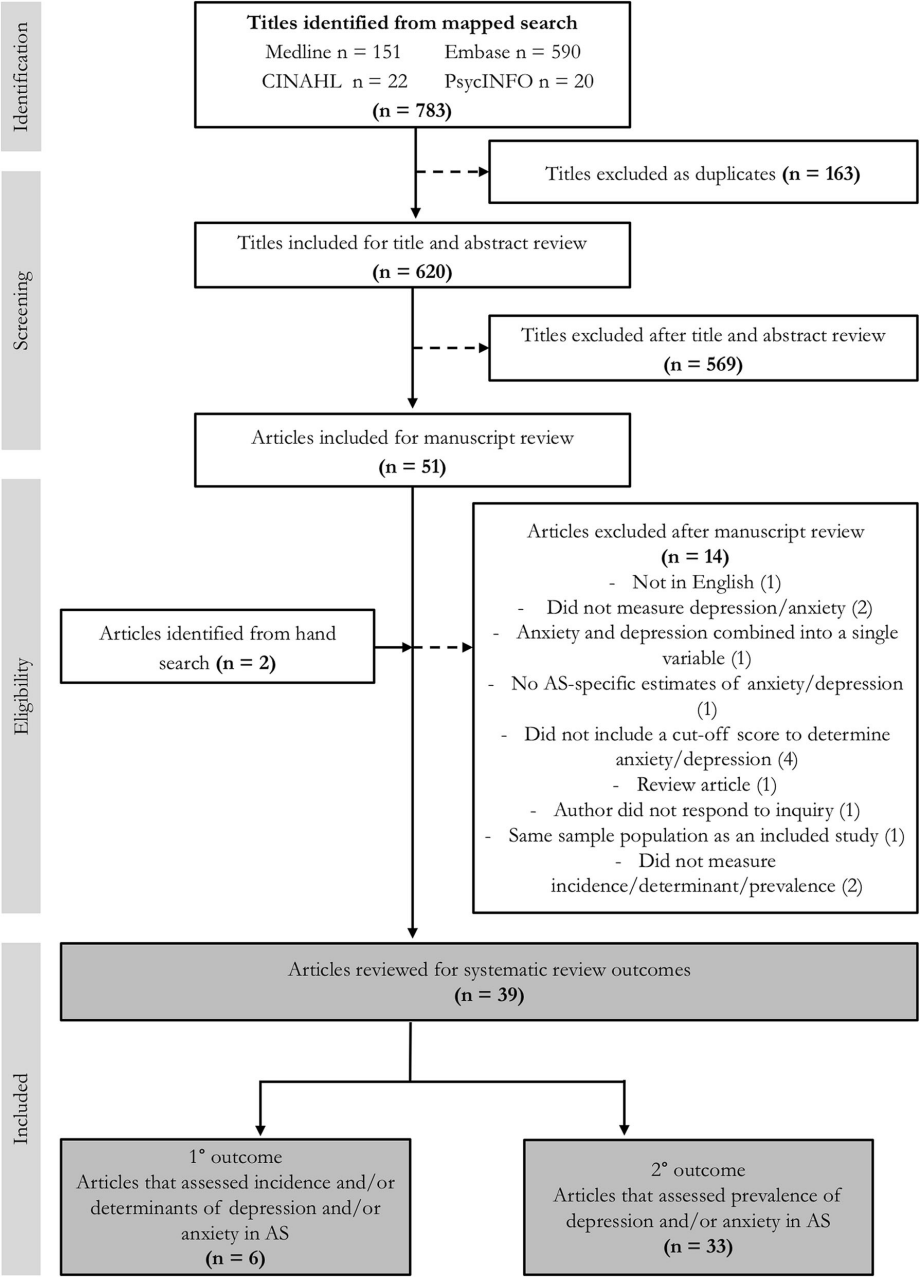
PRISMA flow diagram

We identified 783 studies with our search strategy and as shown in the PRISMA flow diagram in Fig. 1: PRISMA flow diagram. We then reviewed 39 articles and grouped these according to systematic review outcomes. Characteristics of six studies reporting on primary outcomes are summarized in Table 1 (characteristics of 33 studies reporting on secondary outcomes are summarized in Additional file 2). The mean age across study samples ranged from 31.5 to 54.5 years with males comprising the majority of participants in most of the studies (Table 1). There were four cohort studies and two cross-sectional studies with and without comparators (Table 1). Of note, quality assessment using the Newcastle-Ottawa Scale for cohort studies and modified Newcastle-Ottawa Scale for cross-sectional studies resulted in mean scores of 7.8 and 6.0 respectively, suggesting that the included studies were of good quality (Table 1).

**Table 1:** Characteristics of included studies assessing incidence and determinants of depression and/or anxiety in ankylosing spondylitis

Author, year	Country	Study Design	Setting^a^/Data source	Sample Size	Age, mean (SD)	Gender (% males)	AS assessment	Quality assessment score
Wu, 2017 [19]	United States	cohort study	administrative health database	AS: 1878	AS: 52 (16)	AS: 70%	ICD-9	7
				No AS: 156093	No AS: 54 (16)	No AS: 49%		
Shen, 2016 [20]	Taiwan	cohort study	administrative health database	AS: 2331	AS: 36.5	AS: 65%	A-code, ICD-9	8
				No AS: 9324	No AS: 36.5	No AS: 65%		
Zou, 2016 [21]	China	cross-sectional (no comparator)	outpatient	AS: 40	AS: 31.5 (10.1)	AS: 70%	ASAS criteria	6
Kilic, 2014 [22]	Turkey	cross-sectional (w comparator)	outpatient	AS: 174	AS: 38.3	not reported	ASAS criteria	6
				Nr-axSpA: 142	Nr-axSpA: 33.9			
Meesters, 2014 [23]	Sweden	cohort study	administrative health database	AS: 1738	AS: 54.5 (14.3)	AS: 64%	ICD-10	8
				No AS: 967012	No AS: not reported	No AS: 48%		
Sundquist, 2008 [24]	Sweden	cohort study	administrative health database	AS: 5253	Men: 43	AS: 71%	ICD 8–10	8
					Women: 43			

*Abbreviations*: *AS* Ankylosing spondylitis, *ASAS* Assessment of SpondyloArthritis International Society classification, *mNY* modified New York, *ACR* American College of Rheumatology, *ICD* International Statistical Classification of Diseases and Related Health Problems, *Nr-axSpA* Non-radiographic axial spondylarthritis

^a^Settings: Outpatient includes general practitioner medical clinics, rheumatology clinics, community clinics and inpatient includes admissions to a hospital setting

**Fig. 2:**
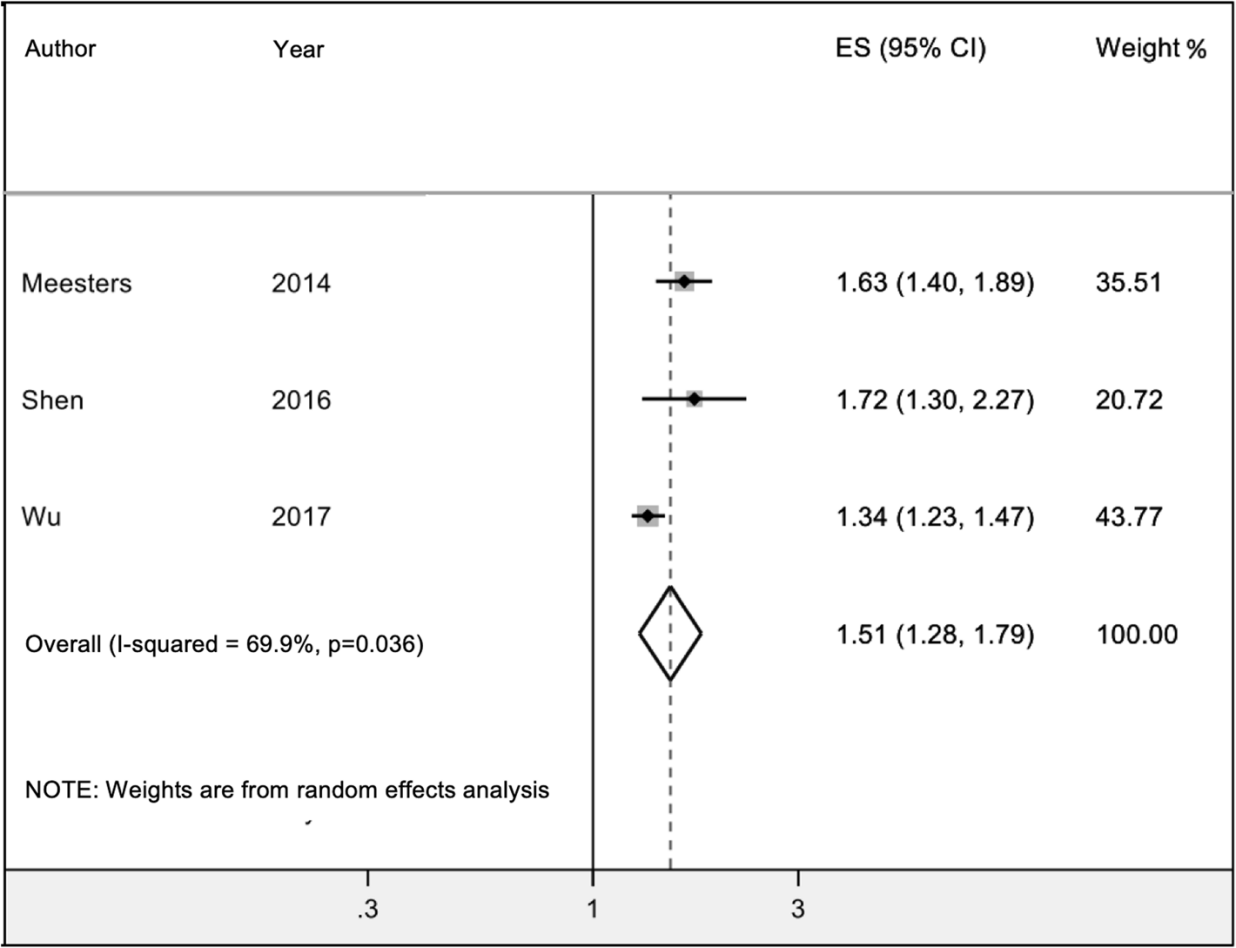
Forest plots representing meta-analyses of incidence estimates for depression among patients with ankylosing spondylitis

Four included studies applied longitudinal cohort designs to administrative databases to assess the risk of depression among patients with AS compared to individuals without AS [19, 20, 23, 24]. Of these, one study additionally assessed the risk of anxiety [20]. Table 2 summarizes the method of outcome assessment and overall reported risk estimates, and when available, risk estimates stratified by gender. The earliest of these studies by Sundquist et al. [24] in 2008 used administrative data from the MigMed database in Sweden and broadly assessed hospitalization for various neuropsychiatric disorders across rheumatic diseases including AS. Authors reported standardized incidence ratios for severe depression of 0.89 (95% CI 0.08 to 2.38) among women with AS and 1.27 (95% CI 0.46 to 2.79) among men with AS compared to the matched general population controls [24]. The latest of these studies by Wu et al. [19] in 2017 used data from two US healthcare organizations and similarly evaluated the risk of multiple mental health issues – including depression – across a number of diseases, including AS. When depression was the outcome in patients with AS, authors reported a hazard ratio (HR) of 1.34 (95% CI, 1.23 to 1.47), adjusted for age, gender, smoking and obesity conditions, comorbidities, outpatient care utilization, enrolment history, and year [19]. With respect to more specific studies, in 2014, Meesters et al. [23] studied the risk of depression among 1738 AS patients as compared with general population controls using administrative data from the Skane Health Register. Authors reported a standardized depression incidence rate ratio (RR) of 1.63 (95% CI 1.40 to 1.89) overall and 1.49 (95% CI 1.20 to 1.89) among men and 1.81 (95% CI 1.44 to 2.24) among women [23]. Of note, authors calculated the ratio between the RR for women and men as 1.20 (95% CI, 0.89 to 1.62) and suggested no substantial interaction between AS and sex for risk of depression [23]. Finally, among included studies, Shen et al. [20] in 2016 specifically evaluated both depression and anxiety among patients with 2331 AS patients and 9324 controls using administrative data from Taiwan’s National Health Insurance Program. Authors reported HR of 1.72 (95% CI 1.30 to 2.27) for depression and 1.85 (95% CI 1.37 to 2.49) for anxiety, after adjusting for age, gender, comorbidities, urbanization, and income [20]. Authors additionally evaluated various factors such as follow-up duration where they showed over 2-fold risk of depression and anxiety beyond the fifth year following AS diagnosis [20]. They also evaluated roles of sex and review of supplementary material requested from authors suggests that both males and females have an increased risk for depression but only males have an increased risk for anxiety (Table 2) [20]. Finally, we extracted data from three studies [19, 20, 23] that reported overall risk estimates for depression for a combined 5947 individuals with AS and conducted meta-analysis to obtain pooled RR of 1.51 (95% CI 1.28 to 1.79) (Fig. 2: Forest plots representing meta-analyses of incidence estimates for depression among patients with ankylosing spondylitis).

**Table 2:** Incidence of depression and/or anxiety in ankylosing spondylitis

Author, year	Outcome	Method of assessment	Overall	Men	Women
Wu, 2017 [19]	depression	ICD 9: 296.2, 296.3, 300.4, 309, 309.1, 311	aRR^c^: 1.34 (1.23–1.47)		
Shen, 2016 [20]	depression	ICD 9: 296.2, 296.3, 300.4, 311	aHR^a^: 1.72 (1.30–2.27)	aHR^d, e^: 1.83 (1.27–2.63)	aHR^d, e^: 1.54 (1.01–2.36)
	anxiety	ICD 9: 300.0, 300.2, 300.3, 308.3, 309.81	aHR^b^: 1.85 (1.37–2.49)	aHR^d, e^: 2.37 (1.64–3.42)	aHR^d, e^: 1.12 (0.65–1.93)
Meesters, 2014 [23]	depression	ICD 10: F32, F33	RR: 1.63 (1.40–1.89)	RR: 1.49 (1.20–1.89)	RR: 1.81 (1.44–2.24)
Sundquist, 2008 [24]	depression	ICD 8, 9, 10; not listed		SIR: 1.27 (0.46–2.79)	SIR: 0.89 (0.08–3.28)

*Abbreviations*: *ICD* International Statistical Classification of Diseases and Related Health Problems, (a) *HR* adjusted hazard ratio (reported in study), *RR* unadjusted rate ratio (reported in study), *SIR* standardized incidence ratios

^a^Adjusted for age, gender, comorbidities (hypertension, diabetes mellitus, dyslipidemia, coronary artery disease, congestive heart failure, chronic pulmonary disease, malignancy), urbanization, and income; ^b^Adjusted for age, gender, comorbidities (hypertension, diabetes mellitus, dyslipidemia, coronary artery disease, congestive heart failure, chronic pulmonary disease, malignancy), urbanization, and income; ^c^Adjusted for age, gender, comorbidities (cardiovascular disease, cardiovascular disease risk, immune disorders, malignancies), obesity conditions, diabetes, smoking, outpatient care utilization, enrollment history, and year. ^d^Adjusted for age, hypertension, diabetes mellitus, dyslipidemia, coronary artery disease, congestive heart failure, chronic pulmonary disease, malignancy, urbanization, and income; ^e^Obtained as supplementary data from study authors

Among included studies, two reported on determinants of depression and/or anxiety among patients with AS, using multivariable regression approaches to evaluate associations [21, 22]. In 2014, Kilic et al. [22] conducted a cross-sectional study among 174 AS patients with axial spondylarthritis from the Erciyes Spondyloarthritis Cohort in Turkey to evaluate the relationship between various clinical and patient factors and depression and/or anxiety as measured by respective versions of the HADS. AS-related factors with independent associations with depression included disease activity as measured with the Bath AS Disease Activity Index (BASDAI; OR 2.48; 95 CI 1.06 to 5.78) and AS Disease Activity Score-C-reactive protein (ASDAS-CRP; OR 2.48; 95% CI 1.06 to 5.78), and quality of life as measured by the AS Quality of Life questionnaire (ASQoL; OR 1.22; 95% CI 1.12 to 1.33) [22]. Authors also reported quality of life as a determinant of anxiety (ASQoL; OR 1.33; 95% CI 1.18 to 1.50) [22]. A sociodemographic factor associated with both depression and anxiety was education level with 6-fold and 9-fold higher odds of depression (OR 6.65; 9% CI 1.36 to 32.51) and anxiety (OR 9.31; 9% CI 1.39 to 62.19) respectively among individuals with AS who are illiterate or completed primary/secondary school as compared to those who completed university [22]. In 2016, Zou et al. [21] similarly conducted a cross-sectional study among AS patients seen in an outpatient hospital in China and showed that smoking history was associated with a 10-fold higher odds of having depression (OR 10.18; 95% CI 1.23 to 84.23) among men.

Finally, given the parameters of our search and screening, we identified 33 studies that assessed secondary outcomes of interest, namely, prevalence of depression and/or anxiety in AS (Additional file 2). With respect to depression, reported prevalence proportions ranged from 0.03 [20] to 0.59 [25] and we obtained pooled prevalence proportion of 0.29 (95% CI 0.17 to 0.43). The prevalence of anxiety ranged from 0.03 [20] to 0.90 [21] and meta-analysis yielded a pooled prevalence proportion of 0.35 (95% CI 0.27 to 0.43).

## Discussion

Our objective was to expand current understanding on the epidemiology of depression and/or anxiety among patients with AS by synthesizing evidence on the risk and determinants of these psychiatric conditions. Altogether, a pooled relative risk of 1.51 (95% CI 1.28 to 1.79), suggesting a 51% higher risk of depression among individuals with AS compared to those without AS, highlights the importance of monitoring and identifying at risk patients during encounters of care. Our systematic review also identified areas where further work is needed. For example, only one included study assessed the risk of anxiety among AS patients [20], thus calling for future confirmatory studies. Limited research on determinants of depression and/or anxiety risk among AS patients is also a gap as these represent potential ways for identifying at patients who may be at risk for these conditions.

To our knowledge, this is the first systematic review on the incidence of depression and/or anxiety among patients with AS, which builds on prior systematic reviews on the prevalence of depression in this patient population [14]. In their 2018 systematic review, Zhao et al. [14] identified 16 studies and reported the prevalence of depression ranging from 11 to 64% and pooled prevalence of 0.29 (95% CI 0.15 to 0.44), which is consistent with secondary outcomes in our current systematic review. The issue of prevalent (or comorbid) depression is particularly important in AS management, given associations with disease activity and functional impairment [14] as well as the diagnosis delay from the symptom onset [26]. Equally important is the onset of depression following AS diagnosis and as we demonstrated in our systematic review, there is a 51% higher risk of depression among AS patients compared to individuals without AS. In addition, the only study assessing the risk of anxiety in patients with AS by Shen et al. [20] reported an 85% increased risk for anxiety, after adjusting for confounding, in comparison to controls. A number of biologic mechanisms may explain this increased risk and evidence suggests that the inflammatory nature of AS could be closely tied to depression and anxiety. As observed in patients with AS [27], individuals with depression and anxiety have also been shown to have elevated levels of proinflammatory cytokines, including tumour necrosis factor alpha and interleukin-6 [28, 29].

Sex-specific risk estimates for depression and anxiety reported [23, 24] or available upon request [20] from three studies allowed us to examine potential differences between men and women. This may be particularly relevant given the predominance of AS among men than in women [4–6] that contrasts the predominance of psychiatric conditions among women [30, 31]. Two of the studies included our review provided unadjusted risk estimates for depression with conflicting results, with one suggesting no increased risk when stratified by sex [24], and the other reporting significantly increased risk for depression among both men and women with AS [23]. Although both of these studies used administrative health data, they differed in the ICD codes used to define depression. Indeed, the study by Meesters et al. [23] that noted significant risk estimates for men and women used a broader set of ICD codes to define depression. In contrast to Sundquist et al.’s [24] insignificant sex-specific risk estimates for severe depression in patients with AS, when the same authors calculated risk estimates for affective disorders (including depression, dysthymia, and depression not elsewhere classified), standardized incidence ratios for men (1.64, 95% CI 1.21 to 2.16) and women (1.82, 95% CI 1.20 to 2.65) reached significance. The third article reported adjusted sex-specific risk estimates for depression and anxiety notably showed that men with AS have more than a two-fold increased risk for incident anxiety whereas women with AS have no increased risk, and further reported that the risk of depression was significant after stratification, but higher among males [20]. Taken together, these findings emphasize the need for future research to clarify the effect of sex on the risk of mental disorders in AS, and highlight the potential for tailored approaches that address gender differences when managing mental health in patients with AS.

We additionally synthesized evidence on determinants of depression and/or anxiety among patients with AS, as these may represent potential targets for intervention or means for identifying high-risk patients. Only two studies were included, based on our a priori definition of a determinant as a factor having independent association with depression and/or anxiety based on multivariable regression methods. Aside from this limited number of studies, it is also important to note that both included studies used cross-sectional designs and as such, temporality cannot be determined. Nonetheless, associations between measures of AS disease severity and depression, and quality of life and both depression and anxiety [22] point to potential impacts on mental health of managing AS. As well, a 10-fold higher odds of depression associated with smoking reported in one study [21] may suggest a modifiable target. Finally, although two of the studies assessing incidence of depression and anxiety indicated factors they adjusted for such as comorbidities, urbanization, income [20], and outpatient care utilization, obesity conditions, smoking [19] – risk estimates for these associations were not reported, precluding the ability to assess whether any of these were potential determinants of depression and/or anxiety.

Strengths and limitations of our systematic review deserve discussion. We collaborated with an information scientist to develop the database search strategies, with the information scientist executing all of the searches. However, the inclusion of relevant studies may have been limited by publication bias as in any other systematic review. We did not consider abstracts given the importance of being able to assess the quality of the included studies. We also did not consider grey literature, although this may not be problematic given the specificity of our topic, that is the incidence of depression and/or anxiety in AS, lends itself to peer-reviewed scientific manuscripts.

Given the demonstrated association between psychiatric complications, namely depression, and disease activity in AS [14], clinical implications of our systematic review also warrant discussion. In particular, our findings suggesting a 51% higher risk of depression among individuals with AS compared to those without AS, highlights the importance of identifying at risk patients during encounters of care. Though our synthesis indicates that evidence on predictors of depression in AS are limited, there is data suggesting AS-related factors (e.g. disease activity) as potential markers for depression risk [22]. Another consideration is the treatment of psychiatric complications in AS, which to our knowledge, has also received very little attention. In their 2016 study on the prevalence of depression and anxiety among patients with rheumatic diseases (*n* = 514) including 44 with AS, Anyfanti et al. also assessed treatment of psychiatric complications and reported considerable under-treatment with less than 10% of AS patients receiving an antidepressant or antianxiolytic [32]. Finally, given the potential role of inflammation in the relationship between AS and depression [27–29], treatments for AS, particularly anti-tumour necrosis factor (anti-TNF) agents, may also impact depression as suggested in prior clinical study [33].

## Conclusions

Altogether, our systematic review provides confirmatory evidence on the increased risk of depression among patients with AS and underscores the need for future research to substantiate the risk of anxiety. Further, synthesizing the determinants of depression and anxiety provides practical information for the identification of patients at risk as well as prevention efforts, which should be priority when caring for patients with AS.

## Supplementary information

**Additional file 1.** Search strategy.

**Additional file 2.** Characteristics of included studies assessing prevalence of depression and/or anxiety in ankylosing spondylitis.

## Data Availability

Not Applicable.

## Abbreviations

AS: Ankylosing spondylitis
ASDAS-CRP: AS Disease Activity Score-C-reactive protein
ASQoL: AS Quality of Life questionnaire
BASDAI: Bath AS Disease Activity Index
CI: Confidence Interval
HADS: Hospital Anxiety and Depression Scale
ICD: International Classification of Diseases
MeSH: Medical Subject Headings
OR: Odds Ratio
PRISMA: Preferred Reporting Items for Systematic Reviews and Meta-Analysis
RR: Relative Risk
