# Supplementary material for: The incidence of depression and anxiety in patients with ankylosing spondylitis: a systematic review and meta-analysis
Source: BMC Rheumatol. 2020 Mar 2;4:12. doi: 10.1186/s41927-019-0111-6 (PMC7050143; doi:10.1186/s41927-019-0111-6)
Supplement: Supplementary file 1 — Additional file 1. Search strategy. [file 41927_2019_111_MOESM1_ESM.docx]

# Additional File 1: Search Strategy

***Medline, Embase, Cochrane Database of Systematic Reviews***

*EBM Reviews - Cochrane Database of Systematic Reviews 2005 to April 4, 2018, Embase 1974 to 2018 April 06, Ovid MEDLINE(R) Epub Ahead of Print, In-Process & Other Non-Indexed Citations, Ovid MEDLINE(R) Daily and Ovid MEDLINE(R) 1946 to Present*

| 1 | exp "Depressive Disorder"/ or "Depression"/ |
| --- | --- |
| 2 | (depressive or depression or melancholia* or "involutional psychosis" or "involutional psychoses" or "dysthymic disorder*" or "premenstrual dysphoric disorder*" or "seasonal affective disorder*" or "seasonal mood disorder*").ti,ab. |
| 3 | 1 or 2 [DEPRESSION] |
| 4 | exp "Anxiety Disorders"/ or Anxiety/ |
| 5 | (anxiety or agoraphobi* or "neurocirculatory asthenia" or "cardiac neuros*" or "hyperkinetic heart syndrome" or "neurotic disorder*" or psychoneurosis or psychoneuroses or neurosis or neuroses or "obsessive compulsive" or OCD or "anankastic personality" or "hoarding disorder*" or "obsessive hoarding" or "panic disorder*" or "panic attack*" or phobic or phobia* or claustrophobi* or "social anxiety disorder*").ti,ab. |
| 6 | 4 or 5 [ANXIETY] |
| 7 | 3 or 6 [MH] |
| 8 | (English or French or German or Spanish).lg. |
| 9 | 7 and 8 |
| 10 | (Animals/ or Animal Experimentation/ or "Models, Animal"/ or (animal* or nonhuman* or non human* or rat or rats or mouse or mice or rabbit or rabbit or pig or pigs or porcine or dog or dogs or hamster or hamsters or fish or chicken or chickens or sheep or cat or cats or raccoon or raccoons or rodent* or horse or horses or racehorse or racehorses or beagle*).ti,ab.) not (Humans/ or (human* or participant* or patient or patients or child* or seniors or adult or adults).ti,ab.) |
| 11 | 9 not 10 |
| 12 | (orial or comment or letter or newspaper article).pt. |
| 13 | 11 not 12 [MH, with limits, human, substantive] |
| 14 | "Spondylitis, Ankylosing"/ or ankylosing spondylitis/ |
| 15 | ("ankylosing spondylitis" or "ankylosing spondyloarthritis" or "ankylosing spondyloarthritides" or "Bechterew’s disease" or "Bechterews disease" or "Bechterew disease" or " Marie-Struempell’s disease" or " Marie-Struempells disease" or " Marie-Struempell disease" or "spondyloarthritis ankylopoietica" or "rheumatoid spondylitis").ti,ab. |
| 16 | ("ankylating spondylitis" OR "ankylopoietic spondylarthritis" OR "ankylopoietic spondylitis" OR "ankylosing spine" OR "ankylosing spondylarthrosis" OR "ankylosis spondylitis" OR "ankylotic spondylitis" OR "bekhterev disease" OR "morbus bechterew" OR "spinal ankylosis" OR "spine ankylosis" OR "spondylarthritis ankylopoietica" OR "spondylarthritis ankylosans" OR "spondylarthrosis ankylopoietica" OR "spondylitis ankylopoetica" OR "spondylitis ankylopoietica" OR "spondyloarthritis ankylopoietica" OR "vertebral ankyloses").ti,ab. |
| 17 | or/14-16 [ANKYL SPONDYLITIS] |
| 18 | 13 and 17 [ANKYL SPONDYLITIS + MH, with limits, human, substantive]  EBM Reviews - Cochrane Database of Systematic Reviews <2005 to April 04, 2018>  [Embase <1974 to 2018 April 06>](http://ovidsp.tx.ovid.com.ezproxy.library.ubc.ca/sp-3.28.0a/ovidweb.cgi?Titles+Display=G%7CS.sh.203%7C1&S=JPFGFPOGGJDDEDNKNCFKEGFBBMBFAA00)  [Ovid MEDLINE(R) Epub Ahead of Print, In-Process & Other Non-Indexed Citations, Ovid MEDLINE(R) Daily and Ovid MEDLINE(R) <1946 to Present>](http://ovidsp.tx.ovid.com.ezproxy.library.ubc.ca/sp-3.28.0a/ovidweb.cgi?Titles+Display=G%7CS.sh.203%7C591&S=JPFGFPOGGJDDEDNKNCFKEGFBBMBFAA00) |
| 19 | remove duplicates from 18  EBM Reviews - Cochrane Database of Systematic Reviews <2005 to April 04, 2018>  [Embase <1974 to 2018 April 06>](http://ovidsp.tx.ovid.com.ezproxy.library.ubc.ca/sp-3.28.0a/ovidweb.cgi?Titles+Display=G%7CS.sh.206%7C1&S=JPFGFPOGGJDDEDNKNCFKEGFBBMBFAA00)  [Ovid MEDLINE(R) Epub Ahead of Print, In-Process & Other Non-Indexed Citations, Ovid MEDLINE(R) Daily and Ovid MEDLINE(R) <1946 to Present>](http://ovidsp.tx.ovid.com.ezproxy.library.ubc.ca/sp-3.28.0a/ovidweb.cgi?Titles+Display=G%7CS.sh.206%7C460&S=JPFGFPOGGJDDEDNKNCFKEGFBBMBFAA00) |
| 20 | 19 use ppez |
| 21 | 19 use oemezd |

***CINAHL Complete***

*Search from 1982 to April 9^th^, 2018*

| S18 | S17  Limiters - Exclude MEDLINE records |
| --- | --- |
| S17 | S12 AND S16 |
| S16 | S13 OR S14 OR S15 |
| S15 | TI ("ankylating spondylitis" OR "ankylopoietic spondylarthritis" OR "ankylopoietic spondylitis" OR "ankylosing spine" OR "ankylosing spondylarthrosis" OR "ankylosis spondylitis" OR "ankylotic spondylitis" OR "bekhterev disease" OR "morbus bechterew" OR "spinal ankylosis" OR "spine ankylosis" OR "spondylarthritis ankylopoietica" OR "spondylarthritis ankylosans" OR "spondylarthrosis ankylopoietica" OR "spondylitis ankylopoetica" OR "spondylitis ankylopoietica" OR "spondyloarthritis ankylopoietica" OR "vertebral ankyloses") OR AB ("ankylating spondylitis" OR "ankylopoietic spondylarthritis" OR "ankylopoietic spondylitis" OR "ankylosing spine" OR "ankylosing spondylarthrosis" OR "ankylosis spondylitis" OR "ankylotic spondylitis" OR "bekhterev disease" OR "morbus bechterew" OR "spinal ankylosis" OR "spine ankylosis" OR "spondylarthritis ankylopoietica" OR "spondylarthritis ankylosans" OR "spondylarthrosis ankylopoietica" OR "spondylitis ankylopoetica" OR "spondylitis ankylopoietica" OR "spondyloarthritis ankylopoietica" OR "vertebral ankyloses") |
| S14 | TI ("ankylosing spondylitis" or "ankylosing spondyloarthritis" or "ankylosing spondyloarthritides" or "Bechterew’s disease" or "Bechterews disease" or "Bechterew disease" or " Marie-Struempell’s disease" or " Marie-Struempells disease" or " Marie-Struempell disease" or "spondyloarthritis ankylopoietica" or "rheumatoid spondylitis") OR AB ("ankylosing spondylitis" or "ankylosing spondyloarthritis" or "ankylosing spondyloarthritides" or "Bechterew’s disease" or "Bechterews disease" or "Bechterew disease" or " Marie-Struempell’s disease" or " Marie-Struempells disease" or " Marie-Struempell disease" or "spondyloarthritis ankylopoietica" or "rheumatoid spondylitis") |
| S13 | (MH "Spondylitis, Ankylosing") |
| S12 | S10 NOT S11 |
| S11 | (PT "letter" OR "commentary" OR "orial" OR "letter" OR "letter to the or" OR "newspaper" OR "pamphlet" OR "pamphlet chapter") |
| S10 | S8 NOT S9 |
| S9 | ((MH "Vertebrates+") NOT MH Human) |
| S8 | S7 AND (LA (english OR french OR german OR spanish)) |
| S7 | S3 OR S6 |
| S6 | S4 OR S5 |
| S5 | TI (anxiety OR agoraphobi* OR "neurocirculatory asthenia" OR "cardiac neuros*" OR "hyperkinetic heart syndrome" OR "neurotic disorder*" OR psychoneurosis OR psychoneuroses OR neurosis OR neuroses OR "obsessive compulsive" OR OCD OR "anankastic personality" OR "hoarding disorder*" OR "obsessive hoarding" OR "panic disorder*" OR "panic attack*" OR phobic OR phobia* OR claustrophobi* OR "social anxiety disorder*") OR AB (anxiety OR agoraphobi* OR "neurocirculatory asthenia" OR "cardiac neuros*" OR "hyperkinetic heart syndrome" OR "neurotic disorder*" OR psychoneurosis OR psychoneuroses OR neurosis OR neuroses OR "obsessive compulsive" OR OCD OR "anankastic personality" OR "hoarding disorder*" OR "obsessive hoarding" OR "panic disorder*" OR "panic attack*" OR phobic OR phobia* OR claustrophobi* OR "social anxiety disorder*") |
| S4 | (MH " Anxiety Disorders+") OR (MH "Anxiety") |
| S3 | S1 OR S2 |
| S2 | TI (depressive OR depression OR melancholia* OR "involutional psychosis" OR "involutional psychoses" OR "dysthymic disorder*" OR "premenstrual dysphoric disorder*" OR "seasonal affective disorder*" OR "seasonal mood disorder*") OR AB (depressive OR depression OR melancholia* OR "involutional psychosis" OR "involutional psychoses" OR "dysthymic disorder*" OR "premenstrual dysphoric disorder*" OR "seasonal affective disorder*" OR "seasonal mood disorder*") |
| S1 | (MH "Depression+") |

***PsycINFO***

*Search from 1597 to April 9^th^, 2018, with comprehensive coverage from the 1880s*

| S14 | S10 AND S13 |
| --- | --- |
| S13 | S11 OR S12 |
| S12 | TI ("ankylating spondylitis" OR "ankylopoietic spondylarthritis" OR "ankylopoietic spondylitis" OR "ankylosing spine" OR "ankylosing spondylarthrosis" OR "ankylosis spondylitis" OR "ankylotic spondylitis" OR "bekhterev disease" OR "morbus bechterew" OR "spinal ankylosis" OR "spine ankylosis" OR "spondylarthritis ankylopoietica" OR "spondylarthritis ankylosans" OR "spondylarthrosis ankylopoietica" OR "spondylitis ankylopoetica" OR "spondylitis ankylopoietica" OR "spondyloarthritis ankylopoietica" OR "vertebral ankyloses") OR AB ("ankylating spondylitis" OR "ankylopoietic spondylarthritis" OR "ankylopoietic spondylitis" OR "ankylosing spine" OR "ankylosing spondylarthrosis" OR "ankylosis spondylitis" OR "ankylotic spondylitis" OR "bekhterev disease" OR "morbus bechterew" OR "spinal ankylosis" OR "spine ankylosis" OR "spondylarthritis ankylopoietica" OR "spondylarthritis ankylosans" OR "spondylarthrosis ankylopoietica" OR "spondylitis ankylopoetica" OR "spondylitis ankylopoietica" OR "spondyloarthritis ankylopoietica" OR "vertebral ankyloses") |
| S11 | TI ("ankylosing spondylitis" OR "ankylosing spondyloarthritis" OR "ankylosing spondyloarthritides" OR "Bechterew’s disease" OR "Bechterews disease" OR "Bechterew disease" OR " Marie-Struempell’s disease" OR " Marie-Struempells disease" OR " Marie-Struempell disease" OR "spondyloarthritis ankylopoietica" OR "rheumatoid spondylitis") OR AB ("ankylosing spondylitis" OR "ankylosing spondyloarthritis" OR "ankylosing spondyloarthritides" OR "Bechterew’s disease" OR "Bechterews disease" OR "Bechterew disease" OR " Marie-Struempell’s disease" OR " Marie-Struempells disease" OR " Marie-Struempell disease" OR "spondyloarthritis ankylopoietica" OR "rheumatoid spondylitis") |
| S10 | S9  Limiters - Language: English, French, German, Spanish |
| S9 | S7 NOT S8 |
| S8 | (PT "letter" OR "commentary" OR "orial" OR "letter" OR "letter to the or" OR "newspaper" OR "pamphlet" OR "pamphlet chapter") |
| S7 | S3 OR S6 |
| S6 | S4 OR S5 |
| S5 | TI (anxiety or agoraphobi* or "neurocirculatory asthenia" or "cardiac neuros*" or "hyperkinetic heart syndrome" or "neurotic disorder*" or psychoneurosis or psychoneuroses or neurosis or neuroses or "obsessive compulsive" or OCD or "anankastic personality" or "hoarding disorder*" or "obsessive hoarding" or "panic disorder*" or "panic attack*" or phobic or phobia* or claustrophobi* or "social anxiety disorder*") OR AB (anxiety or agoraphobi* or "neurocirculatory asthenia" or "cardiac neuros*" or "hyperkinetic heart syndrome" or "neurotic disorder*" or psychoneurosis or psychoneuroses or neurosis or neuroses or "obsessive compulsive" or OCD or "anankastic personality" or "hoarding disorder*" or "obsessive hoarding" or "panic disorder*" or "panic attack*" or phobic or phobia* or claustrophobi* or "social anxiety disorder*") |
| S4 | ((DE "Anxiety Disorders" OR DE "Acute Stress Disorder" OR DE "Castration Anxiety" OR DE "Death Anxiety" OR DE "Generalized Anxiety Disorder" OR DE "Obsessive Compulsive Disorder" OR DE "Panic Disorder" OR DE "Phobias" OR DE "Post-Traumatic Stress" OR DE "Posttraumatic Stress Disorder" OR DE "Separation Anxiety Disorder") OR (DE "Anxiety")) OR (DE "Panic Disorder") |
| S3 | S1 OR S2 |
| S2 | TI (depressive or depression or melancholia* or "involutional psychosis" or "involutional psychoses" or "dysthymic disorder*" or "premenstrual dysphoric disorder*" or "seasonal affective disorder*" or "seasonal mood disorder*") OR AB (depressive or depression or melancholia* or "involutional psychosis" or "involutional psychoses" or "dysthymic disorder*" or "premenstrual dysphoric disorder*" or "seasonal affective disorder*" or "seasonal mood disorder*") |
| S1 | DE "Major Depression" OR DE "Anaclitic Depression" OR DE "Dysthymic Disorder" OR DE "Endogenous Depression" OR DE "Late Life Depression" OR DE "Postpartum Depression" OR DE "Reactive Depression" OR DE "Recurrent Depression" OR DE "Treatment Resistant Depression" OR DE "Seasonal Affective Disorder" |
