# Supplementary material for: The incidence of depression and anxiety in patients with ankylosing spondylitis: a systematic review and meta-analysis
Source: BMC Rheumatol. 2020 Mar 2;4:12. doi: 10.1186/s41927-019-0111-6 (PMC7050143; doi:10.1186/s41927-019-0111-6)
Supplement: Supplementary file 2 — Additional file 2. Characteristics of included studies assessing prevalence of depression and/or anxiety in ankylosing spondylitis. [file 41927_2019_111_MOESM2_ESM.docx]

**Additional File 2: Characteristics of included studies assessing prevalence of depression and/or anxiety in ankylosing spondylitis**

| **Author, year** | **Country** | **Study Design** | **Setting^a^/**  **Data source** | **Sample Size** | **Age, mean (SD)** | **Gender**  **(% males)** | **AS assessment** | **Quality assessment score** |
| --- | --- | --- | --- | --- | --- | --- | --- | --- |
| Jiang, 2018 (1) | China | cross-sectional  (w comparator) | outpatient | AS: 683  No AS: 697 | AS: 27.3 (8.7) | AS: 80%  No AS: 77% | mNY criteria | 6 |
| Sag, 2018 (2) | Turkey | cross-sectional  (w comparator) | outpatient | AS: 50  No AS: 30 | AS: 41.5 (7.8)  No AS: 42.6 (7.9) | AS: 70%  No AS: 67% | ASAS criteria | 6 |
| Oskay, 2017 (3) | Turkey | cross-sectional  (no comparator) | outpatient | AS: 163 | not reported | not reported | mNY criteria | 6 |
| Anyfanti, 2016 (4) | Greece | cross-sectional  (no comparator) | outpatient | AS: 514 | AS: 54.5 (14.3) | AS: 17% | not reported | 6 |
| Shen, 2016 (5) | Taiwan | cohort study | administrative health database | AS: 2,331  No AS: 9,324 | AS: 36.5  No AS: 36.5 | AS: 65%  No AS: 65% | A-code, ICD-9 | 8 |
| Yildirim, 2017 (6) | Turkey | cross-sectional  (w comparator) | outpatient | AS: 51  No AS: 42 | AS: 38.7 (10.4)  No AS: 37.8 (4.6) | AS: 37%  No AS: 24% | ASAS criteria | 6 |
| Zhang, 2016 (7) | China | cross-sectional  (no comparator) | outpatient | AS: 314 | AS: 27.6 (8.3) | AS: 75% | mNY criteria | 7 |
| Zou, 2016 (8) | China | cross-sectional  (no comparator) | outpatient | AS: 40 | AS: 31.5 (10.1) | AS: 70% | ASAS criteria | 6 |
| Bagnato, 2015 (9) | Italy | cross-sectional  (w comparator) | outpatient | AS: 23  No AS: 28 | AS: 51.2 (10.4)  No AS: 48.3 (12.0) | not reported | ACR/EULAR, ASAS criteria | 6 |
| Cooksey, 2015 (10) | UK | population-based cohort study | inpatient and outpatient | AS: 348 | AS: 56 (13) | AS: 77% | Diagnosis of AS | 5 |
| Ramos, 2015 (11) | Spain | cross-sectional  (no comparator) | outpatient | AS: 115 | AS: 41 (11) | AS: 84% | mNY criteria | 6 |
| Kilic, 2014 (12) | Turkey | cross-sectional  (w comparator) | outpatient | AS: 174  Nr-axSpA: 142 | AS: 38.3  Nr-axSpA: 33.9 | not reported | ASAS criteria | 6 |
| Meesters, 2014 (13) | Sweden | cross-sectional  (no comparator) | administrative health database | AS: 571 | 54.5 (13.9) | AS: 65% | ICD-10 | 6 |
| ^b^Shen, 2014 (14)  ^b^Xu, 2016 (15) | China | cross-sectional  (w comparator) | outpatient | AS: 103  No AS: 121 | AS: 32.9 (10.7)  No AS: 37.0 (12.5) | AS: 76%  No AS: 60% | mNY criteria | 6 |
| Hakkou, 2013 (16) | Morocco | cross-sectional  (no comparator) | outpatient | AS: 110 | AS: 38.5 (12.6) | AS: 68% | mNY criteria | 6 |
| Hyphantis, 2013 (17) | Greece | cross-sectional  (w comparator) | inpatient | AS: 55  RA: 199 | AS: 42.9 (10.9)  RA: 55.2 (13.6) | AS: 85%  RA: 18% | mNY criteria | 6 |
| Sariyildiz, 2013 (18) | Turkey | cross-sectional  (w comparator) | outpatient | AS: 70  No AS: 60 | AS: 36.4 (7.4)  No AS: 35.2 (7.7) | AS: 100%  No AS: 100% | mNY criteria | 6 |
| Sariyildiz, 2013 (19) | Turkey | cross-sectional  (w comparator) | outpatient | AS: 37  No AS: 33 | AS: 34.1 (7.0)  No AS: 33.5 (6.2) | AS: 0%  No AS: 0% | mNY criteria | 6 |
| Rezvani, 2012 (20) | Turkey | cross-sectional  (w comparator) | outpatient | AS: 39  No AS: 27 | AS: 38  No AS: 30 | AS: 100%  No AS: 100% | mNY criteria | 6 |
| Rodriguez-Lozano, 2012 (21) | Spain | cross-sectional  (no comparator) | outpatient | AS: 190 | AS: 48.4 (11.7) | AS: 75% | mNY criteria | 6 |
| Baysal, 2011 (22) | Turkey | cross-sectional  (w comparator) | outpatient | AS: 243  No AS: 118 | AS: 34.7 (10.4)  No AS: 36.5 (9.3) | AS: 86%  No AS: 79% | mNY criteria | 6 |
| Healey, 2011 (23) | UK | cross-sectional  (no comparator) | outpatient | AS: 612 | 50.8 (12.2) | AS: 72% | mNY criteria | 6 |
| Ozkorumak, 2011 (24) | Turkey | cross-sectional  (w comparator) | outpatient | AS: 43  No AS: 43 | AS: 36.3 (8.8)  No AS: 36.5 (6.5) | AS: 100%  No AS: 100% | mNY criteria | 6 |
| Kang, 2010 (25) | Taiwan | cross-sectional  (w comparator) | administrative health database | AS: 11,701  No AS: 58,505 | AS:  ≤44: 7,818 (67%)  45–64: 3,275 (28%)  ≥65: 608 (5%)  No AS:  ≤44: 39,090 (67%)  45–64: 16,375 (28%)  ≥65: 3,040 (5%) | AS: 79%  No AS: 79% | ICD-9-CM | 8 |
| Günaydin, 2009 (26) | Turkey | cross-sectional  (no comparator) | outpatient | AS: 62 | AS: 39.6 (10.3) | not reported | mNY criteria | 6 |
| Kobayashi-Gutierrez, 2009 (27) | Mexico | cross-sectional  (no comparator) | outpatient | AS: 18 | not reported | N/A | ACR criteria | 6 |
| Dincer, 2007 (28) | Turkey | cross-sectional  (w comparator) | outpatient | AS: 68  No AS: 45 | AS: 32.9 (11.0)  No AS: 30.1 (6.2) | AS: 100%  No AS: 100% | mNY criteria | 6 |
| Martindale, 2006 (29) | UK | longitudinal study | outpatient | AS: 89 | Median: 50 | AS: 83% | mNY criteria | 4 |
| Karatay, 2004 (30) | Turkey | cross-sectional  (w comparator) | outpatient | AS: 27  No AS: 35 | AS: 51  No AS: 41 | AS: 81%  No AS: 46% | mNY criteria | 6 |
| Pirildar, 2004 (31) | Turkey | cross-sectional  (w comparator) | outpatient | AS: 65  No AS: 65 | AS: 36 (8.1)  No AS: 37 (5.2) | AS: 100%  No AS: 100% | mNY criteria | 6 |
| Barlow, 1994 (32) | England | cross-sectional  (no comparator) | outpatient, local AS self-help groups, symposium | AS: 177 | Male: 43.9  Female: 43.5 | AS: 73% | ACR Criteria | 7 |
| Barlow, 1993 (33) | England | cross-sectional  (no comparator) | outpatient, local AS self-help groups, symposium | AS: 177 | Male: 43.9  Female: 43.5 | AS: 73% | ACR Criteria | 7 |

**Abbreviations:** ASAS: Assessment of SpondyloArthritis International Society classification; NHIRD: National Health Insurance Research Dataset; mNY: modified New York; ACR: American College of Rheumatology; ICD: International Statistical Classification of Diseases and Related Health Problems; EULAR: European League Against Rheumatism; **^a^**Settings: Outpatient includes general practitioner medical clinics, rheumatology clinics, community clinics and inpatient includes admissions to a hospital setting; **^b^**Two separate studies using the same patient sample but they were included because Shen (2014) examined the effect of psychological and physical health while Xu (2016) also investigated predictors of psychological status.

**References**

1. Jiang Y, Yang M, Lv Q, Qi J, Lin Z, Liao Z, et al. Prevalence of psychological disorders, sleep disturbance and stressful life events and their relationships with disease parameters in Chinese patients with ankylosing spondylitis. Clinical Rheumatology. 2018;37(2):407-14.

2. Sag S, Nas K, Sag MS, Tekeoglu I, Kamanli A. Relationship of work disability between the disease activity, depression and quality of life in patients with ankylosing spondylitis. Journal of Back & Musculoskeletal Rehabilitation. 2018:02.

3. Oskay D, Tuna Z, Duzgun I, Elbasan B, Yakut Y, Tufan A. Relationship between kinesiophobia and pain, quality of life, functional status, disease activity, mobility, and depression in patients with ankylosing spondylitis. Turkish Journal of Medical Sciences. 2017;47(5):1340-7.

4. Anyfanti P, Gavriilaki E, Pyrpasopoulou A, Triantafyllou G, Triantafyllou A, Chatzimichailidou S, et al. Depression, anxiety, and quality of life in a large cohort of patients with rheumatic diseases: common, yet undertreated. Clinical Rheumatology. 2016;35(3):733-9.

5. Shen CC, Hu LY, Yang AC, Kuo BI, Chiang YY, Tsai SJ. Risk of Psychiatric Disorders following Ankylosing Spondylitis: A Nationwide Population-based Retrospective Cohort Study. Journal of Rheumatology. 2016;43(3):625-31.

6. Yildirim T, Solmaz D, Emul M, Akgol G, Yalvac D, Ersoy Y. Affective temperament profile in ankylosing spondylitis patients using TEMPS-A. Journal of Physical Therapy Science. 2017;29(3):394-400.

7. Zhang SL, Gao F, Chen ZH, Yan Q, Lin H. Influence of psychological status on back pain and clinical assessments in chinese ankylosing spondylitis patients: A cross-sectional study. International Journal of Clinical and Experimental Medicine. 2016;9(12):23624-33.

8. Zou Q, Jiang Y, Mu F, Shi Y, Fang Y. Correlation of Axial Spondyloarthritis with Anxiety and Depression. Medical Science Monitor. 2016;22:3202-8.

9. Bagnato G, De Andres I, Sorbara S, Verduci E, Corallo G, Ferrera A, et al. Pain threshold and intensity in rheumatic patients: correlations with the Hamilton Depression Rating scale. Clinical Rheumatology. 2015;34(3):555-61.

10. Cooksey R, Brophy S, Dennis M, Davies H, Atkinson M, Irvine E, et al. Severe flare as a predictor of poor outcome in ankylosing spondylitis: a cohort study using questionnaire and routine data linkage. Rheumatology. 2015;54(9):1563-72.

11. Ramos MJM, Ferrando LFL, Martinez MJM. Anxiety and depression in ankylosing spondylitis: A historical view. Revista Colombiana de Reumatologia. 2015;22(4):201-9.

12. Kilic G, Kilic E, Ozgocmen S. Relationship between psychiatric status, self-reported outcome measures, and clinical parameters in axial spondyloarthritis. Medicine. 2014;93(29):e337.

13. Meesters JJL, Petersson IF, Bergman S, Haglund E, Jacobsson LTH, Bremander A. Sociodemographic and disease-related factors are associated with patient-reported anxiety and depression in spondyloarthritis patients in the Swedish SpAScania cohort. Clinical Rheumatology. 2014;33(11):1649-56.

14. Shen B, Zhang A, Liu J, Da Z, Xu X, Liu H, et al. Body image disturbance and quality of life in Chinese patients with ankylosing spondylitis. Psychology & Psychotherapy: Theory, Research & Practice. 2014;87(3):324-37.

15. Xu X, Shen B, Zhang A, Liu J, Da Z, Liu H, et al. Anxiety and depression correlate with disease and quality-of-life parameters in Chinese patients with ankylosing spondylitis. Patient preference & adherence. 2016;10:879-85.

16. Hakkou J, Rostom S, Mengat M, Aissaoui N, Bahiri R, Hajjaj-Hassouni N. Sleep disturbance in Moroccan patients with ankylosing spondylitis: prevalence and relationships with disease-specific variables, psychological status and quality of life. Rheumatology International. 2013;33(2):285-90.

17. Hyphantis T, Kotsis K, Tsifetaki N, Creed F, Drosos AA, Carvalho AF, et al. The relationship between depressive symptoms, illness perceptions and quality of life in ankylosing spondylitis in comparison to rheumatoid arthritis. Clinical Rheumatology. 2013;32(5):635-44.

18. Sariyildiz MA, Batmaz I, Dilek B, Inanir A, Bez Y, Tahtasiz M, et al. Relationship of the sexual functions with the clinical parameters, radiological scores and the quality of life in male patients with ankylosing spondylitis. Rheumatology International. 2013;33(3):623-9.

19. Sariyildiz MA, Batmaz I, Inanir A, Dilek B, Bozkurt M, Bez Y, et al. The impact of ankylosing spondylitis on female sexual functions. International Journal of Impotence Research. 2013;25(3):104-8.

20. Rezvani A, Ok S, Demir SE. Assessment of sexual functions in male patients with ankylosing spondylitis compared with healthy controls. Turkish Journal of Rheumatology. 2012;27(4):233-40.

21. Rodriguez-Lozano C, Gantes MA, Gonzalez B, Hernandez-Beriain JA, Naranjo A, Hernandez V, et al. Patient-acceptable symptom state as an outcome measure in the daily care of patients with ankylosing spondylitis. Journal of Rheumatology. 2012;39(7):1424-32.

22. Baysal O, Durmus B, Ersoy Y, Altay Z, Senel K, Nas K, et al. Relationship between psychological status and disease activity and quality of life in ankylosing spondylitis. Rheumatology International. 2011;31(6):795-800.

23. Healey EL, Haywood KL, Jordan KP, Garratt A, Packham JC. Impact of ankylosing spondylitis on work in patients across the UK. Scandinavian Journal of Rheumatology. 2011;40(1):34-40.

24. Ozkorumak E, Karkucak M, Civil F, Tiryaki A, Ozden G. Sexual function in male patients with ankylosing spondylitis. International Journal of Impotence Research. 2011;23(6):262-7.

25. Kang JH, Chen YH, Lin HC. Comorbidity profiles among patients with ankylosing spondylitis: A nationwide population-based study. Annals of the Rheumatic Diseases. 2010;69(6):1165-8.

26. Gunaydin R, Goksel Karatepe A, Cesmeli N, Kaya T. Fatigue in patients with ankylosing spondylitis: relationships with disease-specific variables, depression, and sleep disturbance. Clinical Rheumatology. 2009;28(9):1045-51.

27. Kobayashi-Gutierrez A, Martinez-Bonilla G, Bernard-Medina AG, Troyo-Sanroman R, Gonzalez-Diaz V, Castro-Contreras E, et al. Depression and its correlation with in patients pain in the rheumatology service of a Mexican teaching hospital. Rheumatology International. 2009;29(10):1169-75.

28. Dincer U, Cakar E, Kiralp MZ, Dursun H. Assessment of sexual dysfunction in male patients with Ankylosing Spondylitis. Rheumatology International. 2007;27(6):561-6.

29. Martindale J, Smith J, Sutton CJ, Grennan D, Goodacre L, Goodacre JA. Disease and psychological status in ankylosing spondylitis. Rheumatology. 2006;45(10):1288-93.

30. Karatay S, Melikoglu MA, Senel K. The relationship between functional disability and depression in patients with rheumatoid arthritis and ankylosing spondylitis. Pain Clinic. 2004;16(4):457-61.

31. Pirildar T, Muezzinoglu T, Pirildar S. Sexual function in ankylosing spondylitis: a study of 65 men. Journal of Urology. 2004;171(4):1598-600.

32. Barlow JH, Macey SJ, Struthers GR. Sex differences and depression in chronic disease patients. In: Dauwalder J-P, Dauwalder J-P, editors. Psychology and promotion of health. Swiss monographs in psychology, Vol. 2. Ashland, OH, US: Hogrefe & Huber Publishers; 1994. p. 210-6.

33. Barlow JH, Macey SJ, Struthers GR. Gender, depression, and ankylosing spondylitis. Arthritis Care & Research. 1993;6(1):45-51.
